# Greenhouse gas released from the deep permafrost in the northern Qinghai-Tibetan Plateau

**DOI:** 10.1038/s41598-018-22530-3

**Published:** 2018-03-09

**Authors:** Cuicui Mu, Lili Li, Xiaodong Wu, Feng Zhang, Lin Jia, Qian Zhao, Tingjun Zhang

**Affiliations:** 1 Key Laboratory of Western China’s Environmental Systems (Ministry of Education), College of Earth and Environmental Sciences, Lanzhou University, Lanzhou, 730000 China; 2 Cryosphere Research Station on the Qinghai-Tibetan Plateau, State Key Laboratory of Cryospheric Science, Northwest Institute of Eco-Environment and Resource, Chinese Academy of Sciences, Lanzhou, Gansu 730000 China; 3 State Key Laboratory of Frozen Soil Engineering, Northwest Institute of Eco-Environment and Resource, Chinese Academy of Sciences, Lanzhou, Gansu 730000 China

## Abstract

Deep carbon pool in permafrost regions is an important component of the global terrestrial carbon cycle. However, the greenhouse gas production from deep permafrost soils is not well understood. Here, using soils collected from 5-m deep permafrost cores from meadow and wet meadow on the northern Qinghai-Tibetan Plateau (QTP), we investigated the effects of temperature on CO_2_ and N_2_O production under aerobic incubations and CH_4_ production under anaerobic incubations. After a 35-day incubation, the CO_2,_ N_2_O and CH_4_ production at −2 °C to 10 °C were 0.44~2.12 mg C-CO_2_/g soil C, 0.0027~0.097 mg N-N_2_O/g soil N, and 0.14~5.88 μg C-CH_4_/g soil C, respectively. Greenhouse gas production in deep permafrost is related to the C:N ratio and stable isotopes of soil organic carbon (SOC), whereas depth plays a less important role. The temperature sensitivity (Q_10_) values of the CO_2,_ N_2_O and CH_4_ production were 1.67–4.15, 3.26–5.60 and 5.22–10.85, without significant differences among different depths. These results indicated that climate warming likely has similar effects on gas production in deep permafrost and surface soils. Our results suggest that greenhouse gas emissions from both the deep permafrost and surface soils to the air will increase under future climate change.

## Introduction

High-mountain environments experience more rapid changes in temperature than those at lower elevations^1^. In the past decades, permafrost degradation accompanying climate warming has been widely detected in mountain permafrost regions as well as in the high-latitude Arctic regions. This degradation is evident from the deepening of the active layer thickness^2,3^, ground temperature increases^4,5^, and thermokarst terrain formations^6^. Permafrost thaws accelerate the rates of carbon and nitrogen released by the soil into the atmosphere and cause a significant positive climate-change feedback^7^. A large amount of soil organic carbon is stored in mountainous permafrost regions, and the carbon pools of these regions are very sensitive to temperature increases^8,9^.

Soil organic matter (SOM) decomposition in permafrost-affected soils is controlled by a complex interplay of environmental parameters such as temperature, soil water content, oxygen and nutrient availability^10^. In particular, temperature has a strong positive effect on aerobic and anaerobic soil respiration rates in permafrost regions^11,12^. Since permafrost degradation typically presents as a gradual increase in soil temperature from below 0 °C, to near 0 °C and then to above 0 °C, incubation experiments using permafrost soils at colder (<0 °C) and warmer temperatures (10 °C)^12–15^ can provide insights into the changes of greenhouse gas release that accompany permafrost degradation.

The Qinghai-Tibetan Plateau (QTP) is the largest low-latitude mountainous permafrost area and has special thermal characteristics; e.g., the ground temperature is considerably higher than that in high-latitude regions, often being near 0 °C^2^. The soil organic carbon (SOC) pools of the QTP have been estimated to approximately 28 Pg in the upper 2 m of soil and approximately 160 Pg for the upper 25 m soils. More than half of the SOC is stored in the soils under meadow and wet meadow^16^. Therefore, obtaining an understanding of greenhouse gas release under these two land cover types is important. The soil respirations in the upper soils, such as the upper 10 cm, has been shown to vulnerable to decomposition and to have a high sensitivity to temperature^17^. However, few studies have been performed to determine the CO_2_ emissions of the deep permafrost on the QTP^6^. Furthermore, the deep permafrost produces unknown amounts of CH_4_ and N_2_O, which are also important greenhouse gases that can be released into the atmosphere^7^.

Permafrost cores can contain a broad range of soil water contents from the active layer to the permafrost table and to the deep permafrost-affected soils^8^. In the permafrost regions on the QTP, the SOM has been well preserved due to the low decomposition rates. Climate warming can stimulate microbial decomposition of the SOM and lead to greenhouse gas emissions under both aerobic and anaerobic conditions. We hypothesized that 1) the production of greenhouse gas, including CO_2_, CH_4_ and N_2_O, of the different permafrost layers is comparable to that in high-latitude permafrost regions throughout the deep soils; 2) the production of greenhouse gas in the deep permafrost is sensitive to temperature increases independent of depth; and 3) the production of greenhouse gas is related to the water-extractable organic carbon (WEOC) content. To test these hypotheses, we collected ~5 m long soil cores from meadows and wet meadows in the permafrost region of the QTP (Table 1) and then measured the greenhouse gas emissions of the soils sampled at different depths of these cores. Soil incubation experiments were conducted under aerobic (to measure CO_2_ and N_2_O production) and anaerobic (to measure CH_4_ production) conditions at temperatures of −2 °C, 5 °C and 10 °C, and the relationships among the soil parameters of the deep permafrost were examined.

**Table 1:** Summary of Borehole Site details.

Site	Latitude(°)	Longitude(°)	Altitude(m)	MAGT(°C)	Aspect	Topography	Land cover	Active layer (m)	Above ground biomass (kg.m^−2^)
#A	98.9627	38.9548	4153	−1.71	Southeast	PS	Meadow	2.0	0.464
#B	98.9630	38.9030	3970	−1.64	Northeast	PSP	Meadow	2.3	0.512
#C	100.9163	37.9979	3691	−0.70 (19 m)	North	PS	Wet meadow	1.4	0.791

MAGT = mean annual ground temperature; PS = piedmont slope; PSP = piedmont sloping plain; The above ground biomass was measured in August 2014 using the harvesting method, and the active layer thicknesses were determined based on monitoring the ground temperatures of the drill holes from 2013 to 2015.

## Results

### Carbon and nitrogen characteristics

**Figure 1:**
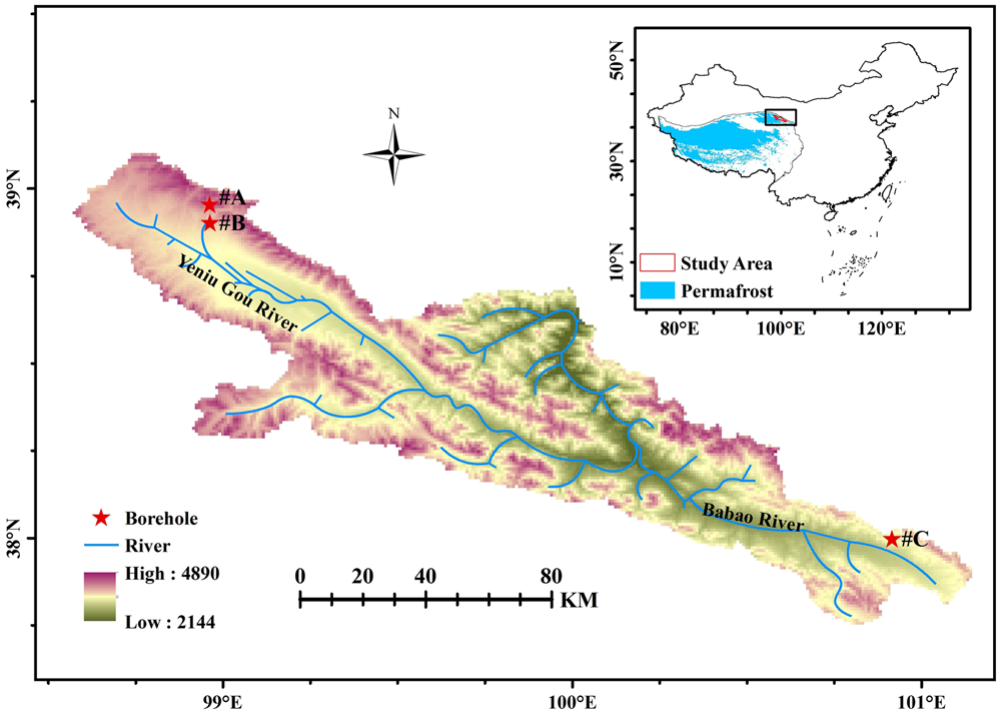
Study area and locations of deep permafrost cores (#A, #B and #C) on the northern Qinghai-Tibetan Plateau. The map was created using ArcGIS 9.3 (https://www.esri.com/en-us/home).

**Figure 2:**
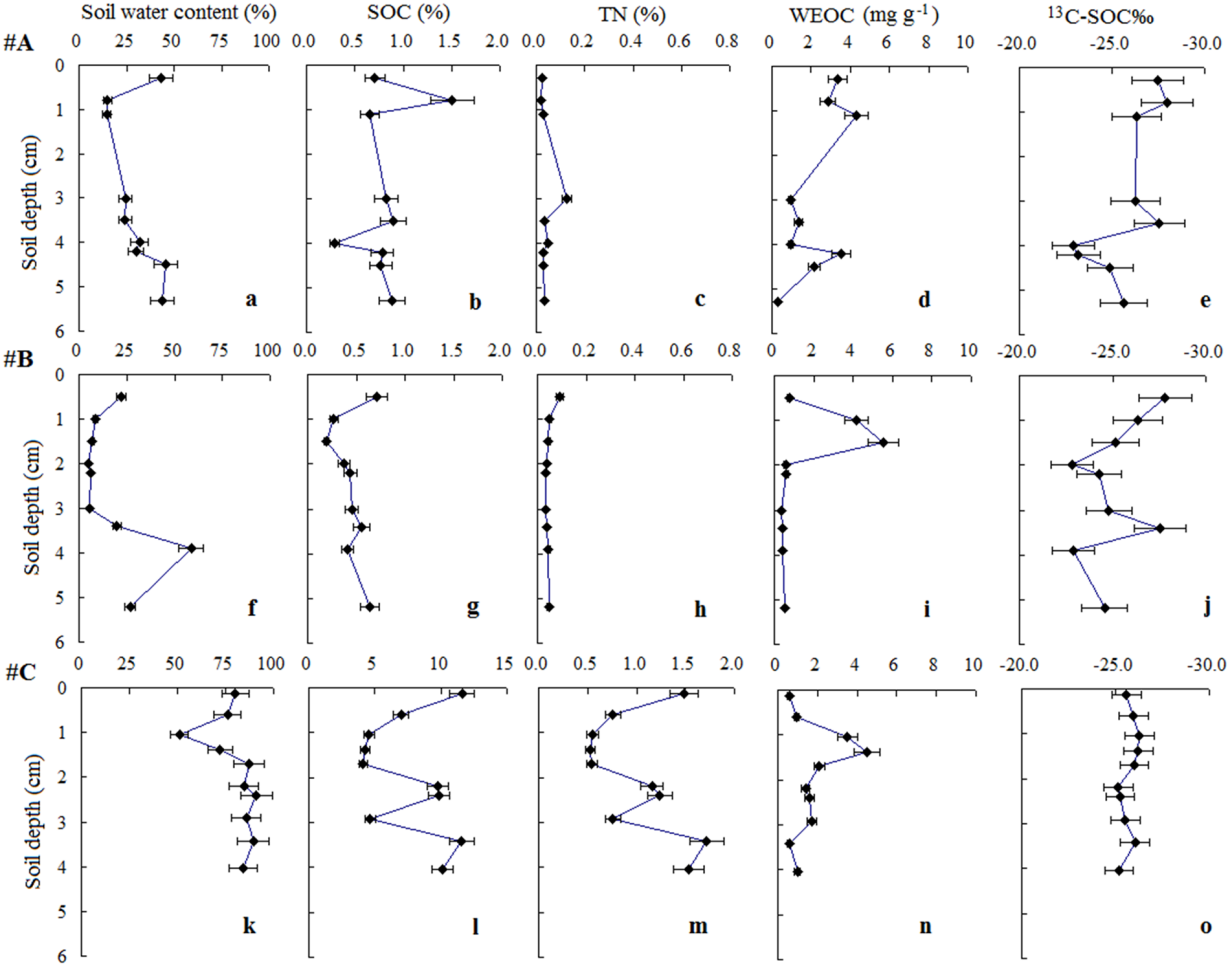
Distribution of soil water content, total nitrogen (TN), soil organic carbon (SOC), water extractable organic carbon (WEOC) and stable carbon isotopes (^13^C-SOC‰) at different depths in #A (a~e), #B (f~j) and #C (k~o).

Soil samples from three deep cores were collected and analyzed (Fig. 1). The deep cores of #A, #B and #C exhibited considerable variations in soil water content, TN, SOC, WEOC and ^13^C-SOC‰ (Fig. 2). Soil water content, TN and SOC content were higher in core #C, which was taken in a wet meadow, than in the cores from meadow (cores #A and #B). Soil water content in cores #A, #B and #C had ranges of 14.7–45.7%, 4.9–58.7%, and 51.2–91.0%, respectively.

The highest values of SOC content among the three cores were observed for wet meadow (4.12–11.6%). The SOC contents of cores #A and #B had ranges of 0.29–1.51% and 0.19–0.72%, respectively. TN content showed patterns similar to those of SOC, with the highest TN content values recorded for wet meadow (0.53–1.72%). For cores #A and #B, the TN contents were typically lower than 0.1%. WEOC content showed patterns different from those of SOC and TN; cores #A, #B and #C had ranges of 0.33–4.29 mg g^−1^, 0.36–5.54 mg g^−1^, and 0.57–4.51 mg g^−1^, respectively. Regarding the stable isotope signatures, the ^13^C-SOC‰ values of deep cores #A (−22.9~−28.0‰) and #B (−22.8~−27.8‰) showed greater vertical changes than did those of core #C (−25.2~−26.3‰).

### Greenhouse gas emissions and their temperature sensitivities

**Figure 3:**
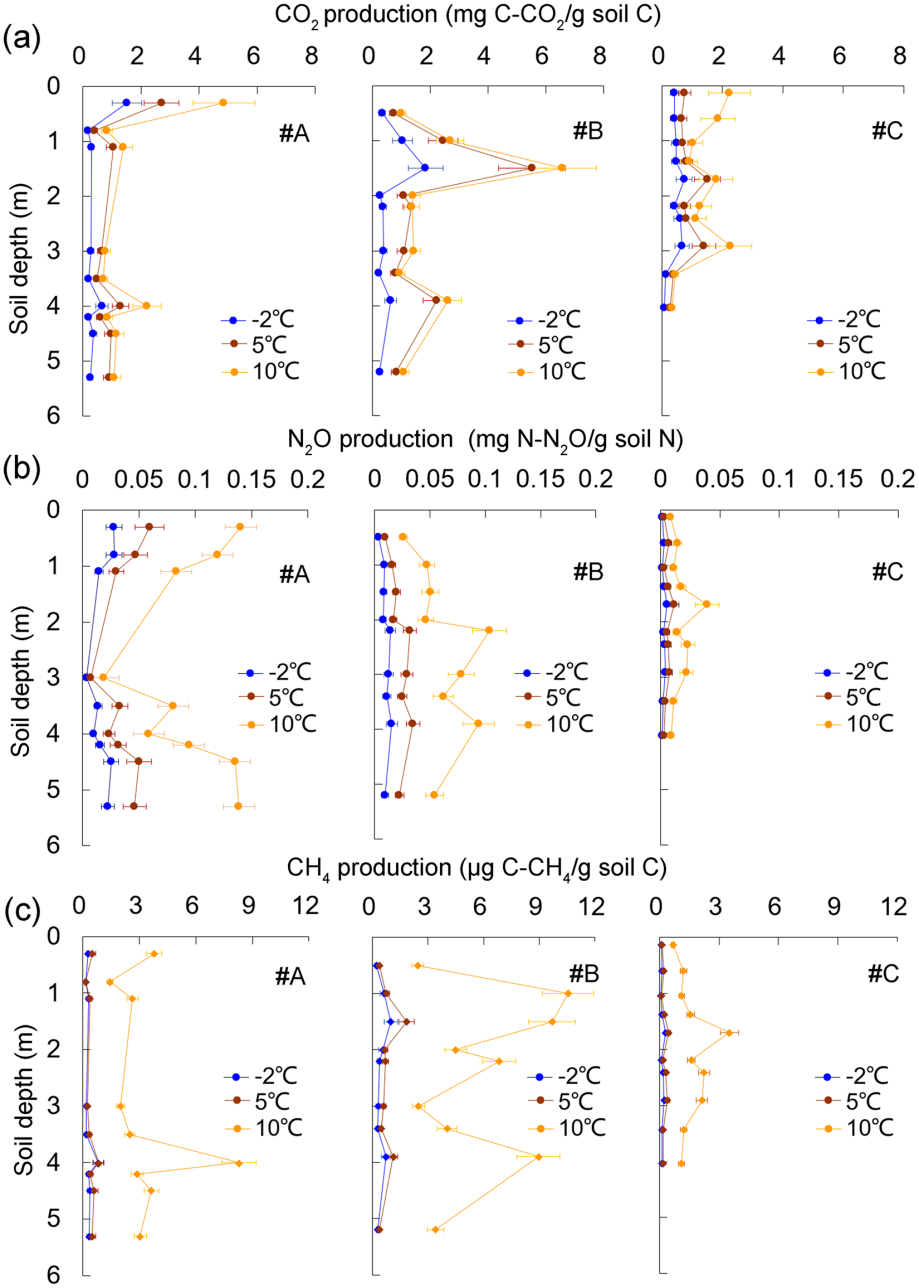
Average CO_2_ (**a**) and N_2_O (**b**) emissions during aerobic incubation and CH_4_ (**c**) emissions during the anaerobic incubation at different depths under −2 °C, 5 °C and 10 °C. The error bars showed the standard deviations (n = 3).

After a 35-day incubation, there were similar trends in the CO_2_, N_2_O and CH_4_ emissions with depth at temperatures of −2 °C, 5 °C and 10 °C (Fig. 3). The production of greenhouse gas was obviously higher at 10 °C than at 5 °C or −2 °C. For core #A, the highest CO_2_ production was 4.86 mg C-CO_2_/g soil C, which was recorded at a depth of 0.3 m. Below this depth, CO_2_ production was typically lower than 2 mg C-CO_2_/g soil C. The highest CO_2_ production was 6.58 mg C-CO_2_/g soil C, observed in soil core #B. For cores #B and #C, the mean CO_2_ production at 10 °C was 2.12 and 1.32 mg C-CO_2_/g soil C, respectively. N_2_O production at the three temperatures showed similar patterns. At 10 °C, the mean values were 0.097, 0.062, and 0.017 mg N-N_2_O/g soil N for cores #A, #B and #C, respectively. The CH_4_ production at −2 °C and 5 °C was similar, but the values increased considerably at 10 °C. The CH_4_ emissions of core #C were lower than those of cores #A and #B. At 10 °C, the mean CH_4_ emissions for cores #A, #B, and #C were 3.36, 5.88, and 1.64 μg C-CH_4_/g soil C.

**Figure 4:**
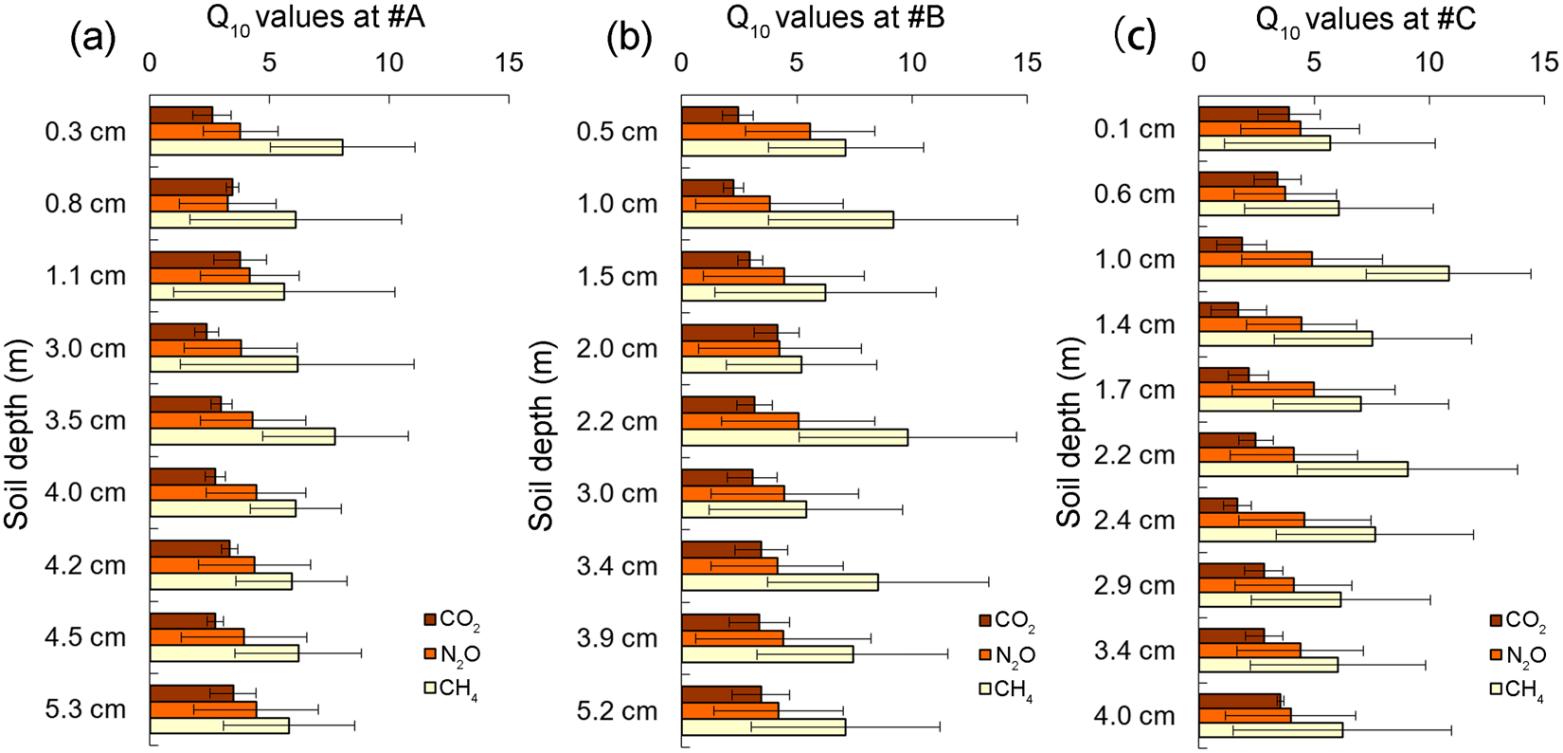
Distributions of the temperature sensitivities (Q_10_) of CO_2_ (**a**), N_2_O (**b**) and CH_4_ (**c**) emissions with depth for cores #A, #B and #C. The Q_10_ values were calculated using the mean greenhouse gas emissions at different incubation temperatures. The error bars showed the standard deviations (n = 3).

The temperature sensitivity (Q_10_) values of the greenhouse gas emissions of cores #A, #B and #C exhibited similar trends, with the Q_10_ values of the CH_4_ emissions being higher than those of the N_2_O emissions and the lowest values observed for the CO_2_ emissions (Fig. 4). The Q_10_ values for the CO_2_ emissions for cores #A, #B and #C had ranges of 2.38~3.79, 2.26~4.15 and 1.67~3.93, respectively. The Q_10_ values of the N_2_O emissions for cores #A, #B and #C had ranges of 3.26~4.44, 3.82~5.60 and 3.76~4.40, respectively. For the CH_4_ emissions of cores #A, #B and #C, the Q_10_ values had ranges of 5.62~7.75, 5.22~9.82 and 5.69~10.85, respectively. For the same greenhouse gas, there were no significant differences among the Q_10_ values at different depths (t-test, p > 0.05, n = 3).

### Factors influencing greenhouse gas production

Across the three cores, there was no significant relationship between depth and production of any of the greenhouse gases. When the greenhouse gas emissions were expressed using a dried soil base, all of the emissions were significantly correlated with soil water, SOC and TN contents (Table 2, supplementary information Dataset 1). When the gas emissions were expressed using a soil carbon base, CO_2_ production was significantly positively correlated with WEOC content, which was negatively correlated with depth. N_2_O and CH_4_ production were both negatively correlated with soil water content, SOC content, and TN content. N_2_O production was significantly correlated with the C:N ratio, and CH_4_ production was significantly correlated with CO_2_ production (Table 2).

**Table 2:** Relationships among soil variables and greenhouse gas emissions (for 10 °C).

	Soil water	^13^C‰	SOC	TN	C:N	WEOC	CO_2_	N_2_O	CH_4_	Depth
Soil water	1.00									
^13^C‰	0.00	1.00								
SOC	0.84**	−0.06	1.00							
TN	0.83**	−0.03	0.99**	1.00						
C:N	−0.31	−0.34	−0.29	−0.36	1.00					
WEOC	−0.16	−0.21	−0.19	−0.20	0.14	1.00				
CO_2_	−0.17	0.06	−0.23	−0.22	−0.15	0.463*	1.00			
N_2_O	−0.49**	0.03	−0.64**	−0.67**	0.68**	0.08	0.16	1.00		
CH_4_	−0.47*	0.38*	−0.55**	−0.50**	−0.20	0.22	0.59**	0.29	1.00	
Depth	0.00	0.46*	−0.16	−0.11	−0.02	−0.42*	−0.34	0.27	0.09	1.00

^*^p < 0.05, **p < 0.01, n = 28. The greenhouse gas emissions were the mean values from the triplicate measurement during the incubation.

## Discussion

### Soil carbon and nitrogen in permafrost layers

In most soils, the SOC and TN contents decrease with depth. Vertical decreasing trends of SOM have been observed in the arid areas of the QTP^18^. In this study, the SOC and TN contents were negatively correlated with depth, but these negative correlations were not statistically significant. This result might be explained by the effects of permafrost on SOM: SOM content can be high in deep soils when the SOM has been protected from microbial decomposition by permafrost^19^. Cryoturbation processes can also bury some organic layers in deep soils^20^.

The ^13^C‰ and WEOC showed significant relationships with depth. WEOC is typically characterized by low molecular weight compounds, which can be directly transported across microbial cell membranes, and thus mainly consists of labile fractions^21^. In addition, the decomposition of organic matter fractionates the isotopic signal of the SOC since the respired CO_2_ must be ^13^C depleted^22–24^. For example, microorganisms may preferentially respire CO_2_ that is ^13^C-depeted relative to the substrate^25^. Therefore, the remaining SOC in deep soils has a more enriched ^13^C-signature. The ^13^C‰ distribution over different depths might also be explained by the significant correlation of WEOC with depth; i.e., the labile C pool (WEOC) decreased with depth, thus causing an increase in ^13^C-signature with depth. The significant relationships between soil water content and each of SOC and TN reflect the fact that a high soil water content limits the decomposition of SOM because it reduces the oxygen availability for microbial decomposition^26,27^.

### Greenhouse gas emissions

Temperature is one of the important controls of organic matter decomposition^6^. In this study, greenhouse gas emissions increased with temperature during both the aerobic and anaerobic incubations. The greenhouse gas emissions in our study were comparable to those observed in circum-Arctic regions and northern China peatlands (Table 3). Based on these findings, the potential release of greenhouse gas via the decompositions of SOM in the QTP can be inferred to be similar to that of high-latitude permafrost regions, although the QTP has lower SOM contents than those in circum-Arctic regions^28^. However, there have been considerable changes in the emissions of greenhouse gas in the QTP, indicating that factors such as temperature^6,17,29^, soil water content^30^ and the chemical characteristics of the SOM^31^ play important roles in the emission of greenhouse gas in permafrost regions. Future studies are needed to determine the quantitative relationships between these factors and greenhouse gas emissions.

**Table 3:** Greenhouse gas emissions of previously published works and our study.

	Area	SOC/TN content	Incubation temperature	Reported emissions	35 day production*	References
CO_2_ emissions	Siberian tundra	5–11% SOC	4 °C	3.5–15 mg C–CO_2_/g C /60 days	2.0–8.8 mg C-CO_2_/g C	Walz *et al*., 2017
	Alaskan tundra	1–16% SOC	15 °C	1–3.5 mg C-CO_2_/g C /500 days	2.0–9.0 mg C-CO_2_/g C	Lee *et al*., 2012
	Northern China peat	22–41% SOC	5 °C, 15 °C	0.5–8 mg C-CO_2_/kg soil/h	1.5–22 mg C-CO_2_/g C	Wang *et al*., 2014
	Qinghai-Tibet Plateau wet meadow	4–12% SOC	5 °C	0.25–2 mgC-CO_2_g C/7days	1.3–10 mg C-CO_2_/g C	Mu *et al*., 2016
	Our study	0.3–11% SOC	−2 °C, 5 °C, 10 °C		0.22–6.6 mg C-CO_2_/g C	
CH_4_ emissions	Alaskan tundra	1–16% SOC	15 °C	0.0007–0.58 mg C-CH_4_/g soil /500 days	0.049–4.06 mg C-CH_4_/g C	Lee *et al*., 2012
	Siberian tundra	5–11% SOC	4 °C	0.05–0.3 g C-CH_4_/g C/60 days	0.03–0.175 mg C-CH_4_/g C	Walz *et al*., 2017
	Our study	0.3–11% SOC	−2 °C, 5 °C, 10 °C		0.14–5.88 μg C-CH_4_/g C	
N_2_O emissions	Greenland wetland	0.05–0.2% TN	7 °C	1–6 ugN-N_2_O /kg soil/h	1–3 mgN-N_2_O/g l N	Elberling *et al*., 2010
	Northern China peat	1.4–1.9% TN	5 °C, 15 °C	0.05–1.5 ugN-N_2_O /kg soil/h	0.004–0.1 mgN-N_2_O/g N	Wang *et al*., 2014
	Our study	0.02–1.5% TN	−2 °C, 5 °C, 10 °C		0.003–1.35 mgN-N_2_O/g N	

^*^The 35 day emissions were calculated from the reported emissions with the assumptions that the production rates were constant. The emissions expressed using the SOC and TN bases were calculated according to the reported SOC and TN contents when the reported emissions were expressed for dry soil weights.

The Q_10_ values in our study confirm that the greenhouse gas production is sensitive to increased temperature. For deep permafrost soils, the average Q_10_ values of the CO_2_ and N_2_O emissions were 2.9 and 4.3, respectively. These values are higher than those (2.0–2.2) observed in the high-latitude peat and fen permafrost regions of northern China^29^. The Q_10_ values of the CO_2_ emissions were similar to those (3.4–6.1) observed in Siberian tundra soils^30^. The Q_10_ values of N_2_O production in our study were in the range (1.5–6.9) of those reported for Canadian agricultural soils^32^. The Q_10_ values of CH_4_ are generally higher than those of CO_2_ although there is large variation (ranging from 1.7 to 28) among different temperature conditions^33^. In an experiment using paddy soils, the Q_10_ value of CH_4_ was approximately 7.4 times higher than that of CO_2_^34^. In this study, the Q_10_ values of CH_4_ production ranged from 5.22 to 10.85 and were higher than the values observed for CO_2_ and N_2_O production. The high Q_10_ values of CH_4_ likely result from several mechanisms. First, CH_4_ oxidation is less sensitive to temperature than is methanogenesis due to its lower optimum temperature such that the CH_4_ emissions show high Q_10_ values^35^. Second, the solubilities of CH_4_ and O_2_ decrease with increased temperature, further limiting the oxidation of methane^36^. Third, higher temperatures promote microbial activity and thus provide more substrates for CH_4_ production^37^. Increasing temperature might also shift the composition of the archaeal community and the pathways of methanogenesis towards hydrogenotrophic methanogenesis^38^. Overall, the greenhouse gas emissions of the deep permafrost soils of the QTP are sensitive to temperature increases.

### Relationships between greenhouse gas emissions and soil variables

The greenhouse gas emissions expressed using a soil mass base were significantly correlated with soil water, SOC and TN contents, indicating that more substrates supply favors the greenhouse gas production^6,17^. We mainly focused on the CO_2_ emissions expressed using a SOC base because these emissions can potentially be used to estimate greenhouse gas emissions based on the global permafrost carbon pools. Consequently, the CO_2_ emissions expressed by a soil C base were negatively correlated with the SOC contents. Although the WEOC represents a labile carbon pool SOC^39^, there was no significant relationship between WEOC content and greenhouse gas production. This result confirms that the mechanisms of microbial decomposition are very complicated^17,40^.

There was a significant positive correlation between CH_4_ production and ^13^C‰, which can potentially be attributed to the different effects of soil depth on CH_4_ production and ^13^C‰. ^13^C‰ was significantly positively correlated with depth (^13^C becomes increasingly enriched with depth), whereas CH_4_ production showed no decreasing trend with depth. Additionally, the acetoclastic hydrogenotrophic pathways of methanogenesis allow for microbial activities that are more independent of the ^13^C‰ values of SOM^41^. Previous studies suggested that the availability of labile substrates is one of the limiting factors for methanogenesis^12,42^. In our study, WEOC was not significantly correlated with CH_4_ production, indicating that methanogenesis can be affected by several factors such as pH, temperature, and sulfate accumulations^43^.

The N_2_O emissions expressed using a dried soil base were significantly correlated with soil water, SOC and TN contents (Table 2). These correlations can be attributed to the SOC’s provision of energy and a matrix for denitrifying bacteria, and ammonium nitrogen and nitrate nitrogen are the substrates of nitrification and denitrification^44^. SOC, TN and soil water contents are typically closely associated with each other^45,46^. In the present study, when the N_2_O emissions were expressed by a soil N base, they were found to be significantly and negatively correlated with SOC, TN, and soil water contents since soil N was set as the denominator. The C:N ratio and N_2_O production were significantly positively correlated with each other, likely because the substrates with high C:N ratios in permafrost regions allow for better SOM preservation such that more biodegradable nitrogen^47–49^ is available for nitrification and denitrification^50^.

In conclusion, this study showed that, for the upper 5 m of soil, the SOM, as expressed by the SOC and TN contents, exhibited no clear decreasing trend with depth. Furthermore, the CO_2_, CH_4_ and N_2_O emissions in the deep permafrost soils were comparable to those in high-latitude permafrost regions, and the greenhouse gas emissions were affected by the labile fractions of SOM. The temperature sensitivities of the CO_2_ emissions ranged from 2.9 to 4.3 across the soil cores, suggesting that the production of CO_2_ in the soils beneath the meadows and wet meadows of permafrost regions is sensitive to temperature increases. The sensitivity of CH_4_ production to temperature was considerably greater than that of CO_2_, which is consistent with previous studies of many soils^34,35,37^. The temperature sensitivity of N_2_O production was within the range of sensitivity values observed for circum-Arctic regions. The greenhouse gas emissions in deep permafrost regions are related to the C:N ratio and stable isotopes of SOC, whereas depth plays a less important role. Our results also showed the SOM below 2 m can contribute to global greenhouse gas emissions in permafrost regions.

## Methods

### Sampling and analysis

In 2012–2014, three permafrost boreholes of various lengths were collected using machine drilling. The cores, #A, #B and #C, were located in the northern QTP, northwestern China (Fig. 1). The area is characterized by an alpine semi-arid climate, has an annual mean precipitation of 433 mm and has a mean annual evaporation of 1,080 mm^51^. The three sites had alpine meadow and alpine wet meadow ground conditions; the dominant species was *Kobresia tibetica Maxim*. The vegetation types, average ground temperatures and other geomorphic features are shown in Table 1.

Cores of the active and permafrost layers, which were up to 4.0~5.3 m in length and had diameters of ~15 cm, were collected. The collected cores were wrapped, labeled, and stored in a freezer at −20 °C and transferred to the laboratory. All frozen cores were cut in half lengthwise. Then, one-half of each core was analyzed for its physical and chemical characteristics, and the other half was incubated under aerobic and anaerobic conditions.

The soil water content was determined by drying the soils at 105 °C for 8 h and measuring the soil weights before and after drying. The SOC, total carbon, and total nitrogen (TN) of the pulverized homogenized samples were quantified by dry combustion using a Vario EL elemental analyzer (Elementar, Hanau, Germany). For the measurement of the WEOC, the soil samples were taken from the −20 °C freezer and put into flasks. The flasks were kept at 4 °C for 24 h to thaw the soils. Then, the WEOC was determined by shaking 20 g of moist field soil with 100 ml of deionized water for 5 h; the suspension was then centrifuged and filtered^52^. The stable carbon isotopes in the SOC were analyzed using an OI Analytical Analyzer (Picarro, California, USA)^6^.

### Incubation experiments

Frozen samples from cores #A, #B and #C were slowly thawed from −20 °C to −2 °C in a refrigerator. The soil samples were incubated at constant temperatures of −2 °C, 5 °C and 10 °C under aerobic and anaerobic conditions. The samples used for the aerobic incubations were weighed, placed into pre-weighed mason jars with airtight lids, and then placed in the incubator (Thermo, USA). For the anaerobic incubations, the headspaces of the anaerobic samples were filled with N_2_. The CO_2_ and N_2_O emissions under aerobic conditions and the CH_4_ emissions under anaerobic conditions were calculated using the changes in the headspace gas concentrations over time (adjusted for headspace volume)^14^. The incubation experiments were performed for 35 days at each temperature, and the gas concentrations were measured on days 7, 14, 21, 28 and 35.

The CO_2_ concentrations were measured with a Licor-7000 infrared gas analyzer (Li-Cor, Lincoln, NE, USA) with nitrogen used as the carrier gas. At each measurement point, three 20 ml headspace samples were collected using a syringe through a rubber septum in the container lid over a 48-hour period and were analyzed for their CO_2_ concentrations. After each measurement, the mason jars were maintained in the open position for 12 hours to allow the headspace CO_2_ to equilibrate with the atmosphere. The CH_4_ and N_2_O concentrations were measured using a gas chromatograph (GC, Agilent 7890 A, Agilent Technologies Inc., Santa Clara, CA). The GC was equipped with a flame-ionization detector (FID) and an electron capture detector (ECD). The FID, ECD and column temperatures were held at 200 °C, 330 °C and 55 °C, respectively. High-purity nitrogen was used as the carrier gas for the FID and ECD systems at a flow rate of 30 and 35 ml/min, respectively. After measuring the CH_4_ and N_2_O concentrations, the jars were flushed using N_2_ for 4 hours to ensure aerobic conditions. During the incubation period, the jars were weighed weekly to determine the amount of water lost through evaporation. When necessary, water was added to bring the soil samples to their initial weights.

### Statistical analysis

The values of Q_10_ were calculated by fitting Equation (1) to estimate of the temperature sensitivities of the greenhouse gas:

1 $$P=A{e}^{BT}$$

where *P* is the rate of greenhouse gas production, *T* is the temperature, *e* is an exponential function, *A* and *B* are fitted parameters, and the Q_10_ was calculated as:

2 $${Q}_{10}={e}^{10B}.$$

The CO_2_ and CH_4_ emissions were expressed based on SOC, and N_2_O emissions were expressed based on TN. We also calculated these greenhouses gas emissions based on dried soil weight (supplementary information Dataset 1). The data analyses were performed in R.3.3.3 (https://www.r-project.org/). The greenhouse gas emissions are presented as mean values and standard deviations, and a one-way ANOVA with post-hoc Tukey’s test was used to compare the Q_10_ values at different depths.

### Data availability

All relevant data are available from the corresponding author upon request.

## Electronic supplementary material

Supplementary information

Dataset 1
