# Supplementary material for: Greenhouse gas released from the deep permafrost in the northern Qinghai-Tibetan Plateau
Source: Sci Rep. 2018 Mar 9;8:4205. doi: 10.1038/s41598-018-22530-3 (PMC5844905; doi:10.1038/s41598-018-22530-3)
Supplement: Supplementary file 1 — Supplementary information [file 41598_2018_22530_MOESM1_ESM.doc]

**Greenhouse gas released from the deep permafrost in the northern Qinghai-Tibetan Plateau**

**Author names:** Cuicui Mu^1,2^, Lili Li, Xiaodong Wu^2*^, Feng Zhang^1^, Lin Jia^1^, Qian Zhao^1^, Tingjun Zhang^1*^

**Affiliation**: ^1^ Key Laboratory of Western China's Environmental Systems (Ministry of Education), College of Earth and Environmental Sciences, Lanzhou University, Lanzhou, 730000, China

^2^ Cryosphere Research Station on the Qinghai-Tibetan Plateau State Key Laboratory of Cryosphere Sciences, Northwest Institute of Eco-Environment and Resource, Chinese Academy of Sciences, Lanzhou, Gansu 730000, China

**Corresponding authors:** X.D. Wu, [wxd565@163.com](mailto:wxd565@163.com), or T.J. Zhang, [tjzhang@lzu.edu.cn](mailto:tjzhang@lzu.edu.cn)

**Corresponding address:** Cryosphere Research Station on the Qinghai-Tibetan Plateau, State Key Laboratory of Cryospheric Science, Northwest Institute of Eco-Environment and Resources, CAS, West Road of Donggang, Lanzhou, 730000, China (X.W.), or College of Earth and Environmental Sciences, Lanzhou University, 222 South Tianshui Road, Lanzhou, 730000, China (T.Z.).

Tel: +8618909408575 (X.W.), or +8615117009464 (T.Z.), E-mail: [wxd565@163.com](mailto:wxd565@163.com) (X.W.) or tjzhang@lzu.edu.cn (T. Z.)

Data set 1 Soil variables at different depths at different sampling sites. The soil water content, water extractable organic carbon content, soil organic carbon content, total nitrogen content, CO_2_, CH_4_ productions expressed by soil C, N_2_O productions expressed by soil N, and CO_2_, CH_4_, N_2_O productions expressed by dried soil weight were shown in the dataset.
